# Utilization of Drivers’ Dynamic Visual Characteristics to Find the Appropriate Information Quantity of Traffic Engineering Facilities on Straight Roads of Grassland Highways

**DOI:** 10.3389/fnins.2022.872863

**Published:** 2022-06-07

**Authors:** Hangtian Li, Songfang Xie, Feng Yang, Yang Lu, Shoulin Zhu

**Affiliations:** College of Energy and Transportation Engineering, Inner Mongolia Agricultural University, Hohhot, China

**Keywords:** grassland highway, traffic engineering facility, information quantity, fixation, saccade

## Abstract

To find the appropriate range of information quantity, we studied how the information quantity of traffic engineering facilities (TEFs) on straight roads of grassland highways affects a driver’s eye movements. We used a combination of survey, statistics, analysis of variance, and the establishment of virtual scene to do this research, and carried out simulated driving tests at six levels (Z0, Z1, Z2, Z3, Z4, and Z5) of TEF information quantity. The driver’s fixation duration, visual search breadth, and glance speed were evaluated in a quantitative way. Results showed that the information quantity had a significant impact on eye movements. It is concluded that the information quantity from 0 to 10 bits/km may cause problems to drivers, whereas the information quantity of 40 bits/km serves as the limit. The information quantity from 30 to 40 bits/km is the appropriate one for TEF on grassland highways.

## Introduction

Traffic engineering facility (TEF) is the general name of various traffic signs and engineering facilities on the highway, which is established to minimize traffic accidents, reduce or eliminate horizontal and vertical interference of highways, maximize traffic capacity, reduce public hazards, and enhance driving safety, based on the principles and methods of highway engineering. TEF containing guidance, instructions, warnings, and other information plays a vital role in the management of the road traffic system. Driving is a complex task that heavily relies on vision (Robinson et al., 1972). Over 80% of information on the road is obtained through visual activities (Lansdown, 1997). The driver needs to continuously perceive, judge, and process all kinds of information during driving, which depends on how the driver interacts with the road traffic system (Jan et al., 2017). Insufficient or overloaded information will affect a driver’s cognition, judgment, decision-making, and driving behavior (Yerkes and Dodson, 1908; Miller, 1956; Sperling, 1960; Wickens, 1992), while imposing a negative impact on the driving safety. So, it is necessary to obtain appropriate information quantity while driving (Du et al., 2008; Wang et al., 2009; Sharples et al., 2016). Grassland accounts for 73.3% of Inner Mongolia’s territory, and most of the roads in use and to be built on the grassland are called grassland highways. Generally speaking, grassland highways present the following features: less change in landform, large viewing distances, monotonous landscape, and inadequate highway traffic engineering facilities. Previous studies revealed that drivers received less visual stimulation when driving on grassland highways, and insufficient information quantity often dragged the driver into an unreasonable monotonous state, which easily resulted in psychological and physical fatigue (Xie et al., 2014). Therefore, the relationship between TEF information quantity and a driver’s eye movements is an issue worthy of study, through which the most appropriate TEF information quantity can be determined on grassland highways.

Recently, numerous relevant studies have been conducted on this issue both at home and abroad. Some scholars discussed the impact of road environment on drivers’ visual behaviors. The visual occlusion technique was developed by Senders et al. (1967) to evaluate attentional demand in driving. Reduced visual attention was considered a main cause of traffic crashes (Crundall et al., 2004). Traffic signs may influence a driver’s recognition (Juan, 2005). A study was carried out on a driving simulator to see how the driver understood the guide sign at the exit of the highway (Arup et al., 2014). The effects of urban expressways road shoulders on the eye movements, heart rate and the lateral position of vehicles were studied (Zhao et al., 2015). Eye-tracking technology was used to study and detect the driver’s responses to roadside traffic signs and advertisements (Darja et al., 2016). Visual factors as perceived by a driver’s eyes were quantified using a visual road environment model (Yu et al., 2019). Some scholars investigated the effects of visual and auditory load and driving task demands on drivers’ eye movement behavior, drivers’ average fixation duration, and variance of fixation duration increased with the increase of visual load and task difficulty (Victor et al., 2005). A study was carried out to analyze the influence of the traffic environment and proficiency on drivers’ workload, and the correlation between eye movement behavior and workload was analyzed (Guo, 2009). The results showed that different traffic environments and driving experiences have a significant influence on drivers’ eye movement behavior and workload. It shows that saccade behavior metrics including saccade duration and saccade average speed can reflect the mental effort and workload of drivers. Some scholars discussed the information amount and threshold values of road traffic sign based on drivers’ visual cognition. The greater the amount of information, the slower the driver’s visual search (Liu, 2005). A study was about the relationship between the information quantity and the visual cognition of traffic guide signs (Du et al., 2008). Researchers found that five or six was the maximum number of road names to display on a single traffic sign based on the study of the driver’s visual search (Di et al., 2011). A dynamic visual cognition experiment was carried out in driving simulation barn with an eye tracker to get drivers’ fixation number and duration when they were reading traffic signs with various information quantities. It is recommended that the highway traffic guide sign information volume should not exceed 5 (Liu et al., 2011). Excessive information contained in traffic signs would impose great pressure on drivers and led to hazardous driving behavior (Guo et al., 2016). Researchers adopted the simulation test, designed combined signs containing guide sign and lane directional sign, measured visual cognition accuracy and reaction time, and obtained the information threshold, while combined signs with simple guiding graphics shall be set as 6 pieces, whereas the threshold for more complicated guiding graphics shall be set as 5 pieces (Liu et al., 2016). Researchers investigated drivers’ visual cognition patterns such as eye movement time, saccade frequency, and seek time, regarding exit guide signs on freeway interchanges. It was found that the corresponding threshold values of destination information are 5, 5, 4, and 3 under the four levels of route information, and the threshold value of route information is 3 (Ren et al., 2019). In addition, some scholars discussed the driver factor to compare visual search strategies for road information. Eye movements were used to study a driver’s perception of danger, and it was concluded that a novice had lower levels of perception compared to an experienced driver (Pradhan et al., 2005). A study suggested how efficiently visual search strategies indicated the driver’s transition from a novice to a veteran (Underwood, 2007). The coach and the learner’s eye movements on three types of virtual roads were studied through a driving simulator in the daytime, at night, and in rainy days (Konstantopoulos et al., 2010).

Based on the above comprehensive literature reviews, previous studies provided a theoretical basis for this study and most studies mainly focused on the quantity of traffic sign information, whereas fewer studies focused on the overall quantity of traffic engineering facility information and especially grassland highways. Aiming at the improper setting of traffic engineering facility on grassland highways, it is necessary to study drivers’ visual recognition of the quantity of information contained in traffic engineering facility and explore the appropriate information quantity on straight roads of grassland highways. Therefore, the information quantity of traffic engineering facility was taken as the main control variable, and the TEF information was quantified and classified. Then, a driver’s eye movements were collected at different information quantity levels through a simulation experiment. Meanwhile, the driver experience factor was also considered. By doing so, it is expected to provide theoretical guidance for the design of traffic engineering facility located on grassland highways, and enhance traffic safety.

## Materials and Methods

This section consists of two parts. (1) Based on related information theory, the information quantity model is established to quantify the TEF of grassland highways and classify the information quantity levels. (2) The simulation experiment is designed to collect drivers’ eye movements metrics.

### Quantitation of Information

The information about the road traffic environment system was conveyed by highway TEF to road users with specific shapes, colors, graphics, characters, and positions. The TEF chosen included traffic signs, guidance facilities, sight induction facilities, intersection prompt facilities, and line marking, while had a direct impact on drivers. The information of TEF on the highway describes the attribute state of each component of TEF. There are many components of TEF on the highway, and all the information constitutes a complex and discrete information field. Information theory was put forward by Shannon for the first time (Shannon, 1948). Existing methods to quantify TEF information are mainly based on the Shannon information theory. Hu (2012) applied the Shannon information theory to calculate the quantity of information contained in TEF. Information theory was expanded in the TEF and the formula of TEF information quantity was derived, as shown in the following equation:


(1) $$H\,\left(X\right)=-\sum_{i=1}^{m}P\,\left(X_{i}\right)\,\log_{2}P\,\left(X_{i}\right)$$

where H(X) refers to the quantity of information contained in TEF (unit: bits); Xi refers to the ith state in the event X; m refers to the total number of possible states for the event X to occur; and P(Xi) refers to the probability of the ith state to occur.

It is assumed that each state has the same occurrence probability in each component of TEF, such as words, colors, and graphics, i.e., P(Xi) = 1/m. The calculation of information quantity can be simplified to the following equation:


(2) $$H\,\left(X\right)=\log_{2}m$$

The research object of this study is the typical TEF on the secondary road of grassland highway in Inner Mongolia, so TEF also contains Mongolian words. This study referred to Road Traffic Signs and Marking (GB5768-2009), Highway Design Guidance Manual for Traffic Engineering Facilities, Place Name Signs (GB17733-2008), and the survey on grassland highways. It is generally believed that TEF on the grassland highway contained 9 types of information, including Chinese characters, English letters, Arabic numerals, geometric shapes, colors, directions, diagrams or feature symbols, and Mongolian and linear infrastructure. Referring to relevant standard and manual, 8 types of information components were used which frequently contained 3,500 Chinese characters, 26 English letters, 10 Arabic numerals, 8 geometric shapes, 12 colors, 30 directions, approximately 50 diagrams or feature symbols, and 29 Mongolian letters, respectively, in the TEF. The probability of occurrence P(Xi) of the 8 types of information components, i.e., 1/3,500, 1/26, 1/10, 1/8, 1/12, 1/30, 1/50, and 1/29, can be considered, respectively. In accordance with Equation 2, the single quantity transmitted by each information component is11.77 bits, 4.7 bits, 3.32 bits, 2.58 bits, 3.58 bits, 4.91 bits, 5.64 bits, and 4.86 bits. The linear infrastructures, such as traffic marking, followed the alignment of the highway, while the probability to occur was considered to be the proportion of the total length of a linear infrastructure continuous to the total length of the test highway. According to the above result, the basic information quantity (BIQ) of each type of information is shown in Table 1.

**TABLE 1:** BIQ and coefficient of 9 types of information on grassland highways.

Information type	Chinese characters characters	English	Number	Geometrical shape	Color
BIQ (bits)	11.77	4.70	3.32	2.58	3.58
Weight coefficient	0.21	0.06	0.16	0.11	0.10

**Information type**	**Direction**	**Figure Symbol**	**Mongolian**	**Linear infrastructure**	**Total**

BIQ (bits)	4.91	5.64	4.86	Calculate as actual length ratio	1.00
Weight coefficient	0.17	0.08	0.05	0.06	1.00

Considering that different types of TEF information on grassland highways have different significance, the analytic hierarchy process was adopted (Li, 2017). Li established the hierarchical goal decision level and index set to evaluate the importance degree of 9 types of information element present in the TEF. A total of 15 participants were invited to evaluate the importance degree of 9 types of information element, including 5 experts and scholars engaged in research on the road traffic system, 5 road engineering design personnel and 5 professional drivers. Based on the method of 9 point scale, each evaluation index sequence was calculated. Each type of TEF information on grassland highways is given a weight, as shown in Table 1.

The effective information quantity (EIV) is calculated as follows:


(3) H(E)=εHi(X)ini

where H(E) is the effective information quantity of TEF on grassland highways, or bits; εi is the weight coefficient of the information; H(Xi) is the basic information of the ith piece of information; and ni is the quantity of the ith piece of information in TEF.

It is currently considered that all highway traffic facilities are composed of the above 9 types of information. In accordance with the definition of information theory, there are both self-information and mutual information in this information field, and the information provided to road users is a concept of “and” (“U”). Hence, the traffic information of a highway is composed of the sum of the above information (Hu, 2012).

The following equation gives the total effective TEF information on grassland highways:


(4) $$H=\sum b_{j}\,H\,\left(E_{k}\right)$$

where H is the total effective information quantity of TEF on grassland highways, or bits; H(Ek) is the effective information quantity of the kth TEF, or bits; and bj is the jth TEF quantity.

**FIGURE 1:**
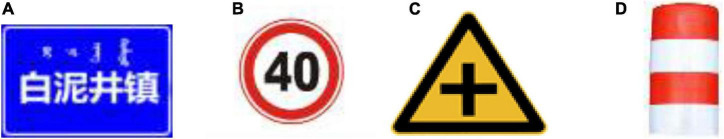
**(A)** Indication sign of geographic location, Bainijing Towns. **(B)** Ban sign of 40 km speed limit. **(C)** Intersection warning sign. **(D)** Warning column.

In this study, the aforementioned computational method was adopted to quantify information contained in TEF on grassland highway. The effective information quantity (EIQ) of each TEF was calculated using Equation 3. For example, it is found that the information quantity of the four traffic signs (A–D) illustrated in Figure 1 amounted to 11.86, 2.42, 1.83, and 1.0 bits, respectively.

To evaluate information contained in TEF on grassland highway accurately, it is necessarily classified according to information quantity. Li (2017) investigated 3 section roads of S101, S309, and S203 in Inner Mongolia, and the total length was nearly 350 km. The total EIQ of TEF was calculated using Equation 4 on surveyed grassland highways for the study. The survey result showed that the type, distribution, and density of TEF were different and dominated by instruction information. The calculation result of information quantity contained in TEF shows that the single information quantity mainly falls in the range of 0–10 bits, up to 30.84 bits on grassland highways of EIQ. However, there may be several and consecutive signs and facilities, and the total information quantity was overloaded within short time. Then, considering this case, the information quantity density was calculated, and it concentrated from 0 to 10 bits/km, while it falls in the average range of 0 to 25.67 bits/km, up to 43.39 bits/km. This further proves that the overall information quantity of TEF on grassland highways was insufficient and not homogeneous. The grassland highway surveyed was divided by a 1 km section to classify the information quantity level. The density of information quantity of straight roads on grassland highways can be divided into (0–10 bits/km), (10–20 bits/km), (20–30 bits/km), (30–40 bits/km), and (over 40 bits/km) (Li, 2017).

### Simulation Experiment

#### Apparatus

**FIGURE 2:**
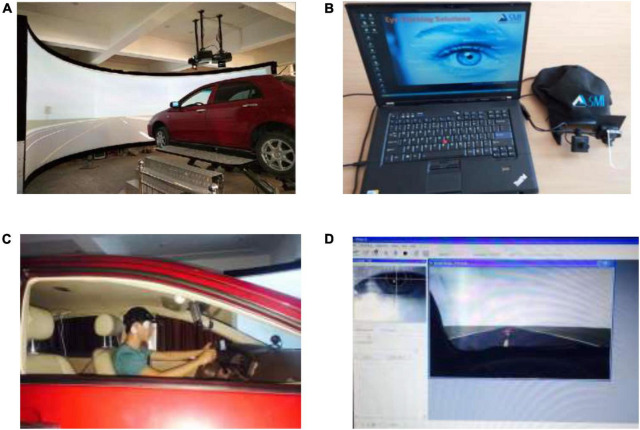
Major apparatus. The human image in **(B)** is from the Eye tracker software. The human image in **(C,D)** is from the tested subject. **(A)** The driving simulation platform. **(B)** Eye tracker. **(C)** The tested subject. **(D)** Eye tracker recording interface.

The driving simulation system platform consists of the vehicle cockpit, 300-degree visual simulation system, vehicle simulation computer, six-degree freedom motion platform, and scene control real-time simulation software. The platform was used to create the scene of grassland highways, control vehicle operation and collect data of the driving behavior. Meanwhile, eye tracker models for iView X (HED) were used to collect the data of driver’s eye movements. The driving simulation platform and the eye tracker are shown in Figure 2. The parameter of eye tracker models for iView X (HED) is shown in Table 2.

**TABLE 2:** Eye tracker parameter.

Models	Weighs	Sampling frequency	Fixation position accuracy	Horizontal tracking range	Vertical tracking range
iView X (HED)	450 g	200 Hz	0.5–1.0°	−30°∼+30°	−25°∼+25°

#### Participants

Human is a key factor in the road traffic system during the driving process. A person’s trait, experience, gender, age, and education background can all affect driving (Tay et al., 2003; Mannering, 2009; Cristea et al., 2013). For the purpose of this study, the driving experience was a key factor for levels of perception (Pradhan et al., 2005; Underwood, 2007), and other factors are kept the same as much as possible. According to the data of national motor vehicles and drivers publicized by the Ministry of Public Security in 2021, the ratio of male to female drivers was 1.97:1, and most private car drivers were male. To control the influence of gender factors on experimental results, 40 male subjects were selected to take the simulated driving test, whose driving year ranged from 2 to 15 years (Mean = 7.7, SD = 3.2), and whose age ranged from 20 to 38 years (Mean = 27.6, SD = 6.2). All the subjects were healthy, and their visual acuity or corrected visual acuity was above 5.0. They all had legal Chinese driving licenses. Through a comprehensive analysis of subjective questionnaire, basic information collection, reaction speed, vehicle control ability, and other tests the drivers were divided into skilled (20 male) and unskilled (20 male) groups. Drivers’ basic data is shown in Table 3.

**TABLE 3:** Drivers’ basic data.

Number	Age	Driving years	Driving range (10^4^ km)	Reaction time for signs/s	Recognition accuracy	average free speed (km/h)
Skilled group	34.4 ± 4.5	5.8 ± 2.5	5.04 ± 4.03	3.69 ± 0.87	0.85 ± 0.06	78.4 ± 12.7
Unskilled group	26.4 ± 4.1	2.2 ± 0.5	1.84 ± 0.93	4.46 ± 1.39	0.83 ± 0.04	67.2 ± 9.3

#### Scene Design

**FIGURE 3:**
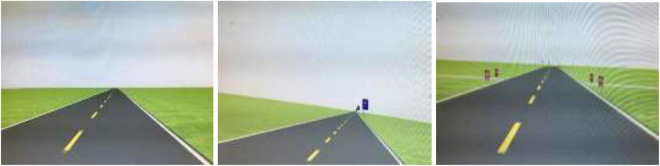
Road scene.

Based on the initial classification of TEF information quantity, the corresponding experimental scene was established on the simulated driving system platform. The basic scene, which was designed according to typical grassland highway road parameters, was close to the actual road environment. This study referred to Road Traffic Signs and Marking (GB5768-2009), Highway Design Guidance Manual for Traffic Engineering Facilities, Place Name Signs (GB17733-2008), and the survey on grassland highways. The straight road was designed to be a typical second-level grassland highway, including two-way lanes with a width of 3.75 m. The driving speed was designed to be 80 km/h and there was no traffic flow. Common TEF information on grassland highways was used. Four types of facilities selected were information of warning signs, ban signs, guide signs, and crossing markers. Parameters such as location, height, and size were set according to regulations and criteria. The information quantity is the only variable in this experiment. The road scene is shown in Figure 3.

There are five levels of information quantity, Z1 (0–10 bits/km), Z2 (10–20 bits/km), Z3 (20–30 bits/km), Z4 (30–40 bits/km), and Z5 (over 40 bits/km), while Z0 (0 bits/km) was set as the control group. The road length for each level was a 5 km straight line, and a typical single sign was set up for every 1 km, respectively, in the same level of information quantity, while avoiding the types effect of various signs. For the purpose of avoiding monotony of the continuous straight line, the transition curve (0.5 km) was set at the end of every two levels. For reducing the impact of the start and end driving, an adaptive straight road (0.5 km) was set at the start and end section of the experimental scene. Therefore, the total length was 32 km. Typical TEFs appeared frequently were selected, and their information quantity was calculated, and according to the various information quantity level, single or combination signs were set up randomly in each section. Parameters for each level are shown in Table 4.

**TABLE 4:** Parameters.

Level	Parameters	Typical TEF	Information quantity /(bits)
Z0	Basic marking	None	0
Z1	Z0+ typical single sign in every 1 km, respectively (such as instruction, warning and limiting-velocity sign)	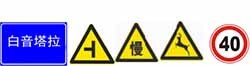	0–10
Z2	Z0+ typical combination signs in every 1 km, respectively (such as instruction and warning signs)	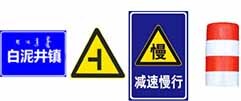	10–20
Z3	Z0+ typical double combination signs in every 1 km simultaneously (such as instruction and warning signs)	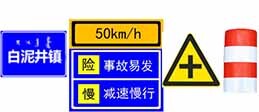	20–30
Z4	Z0+ typical triple combination signs in every 1 km simultaneously (such as instruction and warning signs)	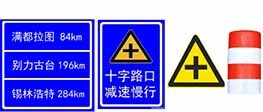	30–40
Z5	Z0+ typical combination signs in every 1 km simultaneously (such as triple instruction, warning and limiting-velocity signs)	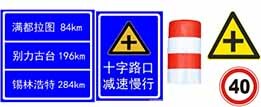	>40

#### Procedures

Before the test, subjects were given sufficient time to rest. All of them took no stimulant drugs. They were trained for more than 30 min on the simulated platform. As a previous study revealed that different times of the day have an influence on the severity of the crash and its rate (Clarke et al., 2006), to eliminate the interference of illuminance on a driver’s vision, the task was done during the day. All tests were conducted within the same time period on adjacent dates. The time period for the test was from 8:00 to 12:00 a.m. and from 2:00 to 6:00 p.m., to reduce the impact of human biological rhythms.

The subject’s fixation point was calibrated as required. Subjects took the task one after another, in which they drove through six sections (each section being 5 km) of the road with different levels of TEF information quantity. While driving, their eye movements were recorded. The experiment kept repeating until all subjects completed their own task.

#### Data Collection and Extraction

Eye movements were collected and recorded at six sections as a driver was exposed to different levels of TEF information quantity, and then the data were processed by the Begaze2.4 software that was matched with eye trackers. The eye movement includes fixation, saccade, and blink. There are many evaluation indices for the eye movement (Underwood, 2007; Li, 2017). Based on the above results and the meaning of the eye movement index, fixation duration, dispersion X, dispersion Y, and saccade average speed were selected to evaluate a driver’s dynamic visual characteristics as the driver was exposed to different levels of TEF information quantity. Fixation duration is the time period in fixated area of interest, represents the time that drivers process traffic information in this study, and reflects the difficulty of information extraction. Dispersion X and dispersion Y are the distribution deviation of fixation point coordinates X and Y, and reflect the quantity of information extraction. Saccade average speed is fast movement speed of the eyes that changes the point of fixation, and reflects the efficiency of information capture and processing.

## Results

The variable was the information quantity on straight roads. Z1 to Z5 were five levels of information quantity, and Z0 was the control group. The difference between two groups (skilled and unskilled) was the driving experience. When a driver was exposed to different levels of information quantity, their eye movements were collected during the 5 km of driving. The eye movement index was averaged for every 1 km, and then the overall value was averaged for every 5 km. This study analyzed how TEF information quantity affects a driver’s eye movements while driving along straight roads on grassland highways.

Frequency statistics of eye movement indices were calculated for all samples. In addition, each index was applied the single sample non-parametric test, namely Kolmogorov-Smirnov (K-S), and it was found that *p*-values were greater than the significance level of alpha = 0.05. This result shows that the driver’s eye movement indices are accord with normal distribution when the driver is exposed to different levels of information quantity. The mean of each eye movement index at different levels of information quantity is shown in Table 5.

**TABLE 5:** Descriptive statistics.

Index level	Z0 (control group,0 bit)	Z1 (0–10 bit/km)	Z2 (10–20 bit/km)	Z3 (20–30 bit/km)	Z4 (30–40 bit/km)	Z5 (over 40 bit/km)
Fixation duration (ms)	313 (32)	368 (67)	344 (62)	336 (51)	325 (86)	369 (31)
Dispersion X (°)	13.1 (1.1)	13.4 (0.7)	16.1 (1.9)	15.5 (1.5)	18.9 (1.8)	14.1 (2.4)
Dispersion Y (°)	33.3 (5.9)	36.1 (3.4)	34.7 (2.4)	37.8 (7.9)	43.4 (7.1)	32.1 (9.4)
Saccade average speed (°/s)	422 (19)	428 (38)	427 (10)	354 (68)	373 (58)	272 (30)

**FIGURE 4:**
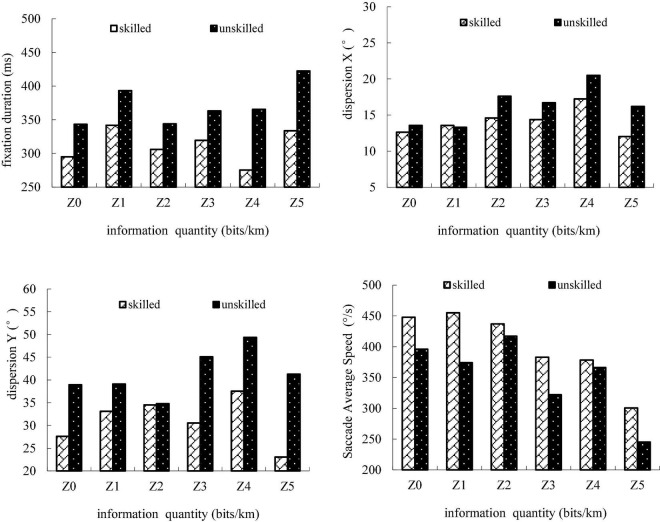
Eye movement of skilled and unskilled drivers.

Figure 4 shows that there is a trend of four metrics and a difference among Z0–Z5 between skilled and unskilled drivers. Two groups of drivers show significantly different eye movement characteristics when driving on straight roads. The fixation duration index of unskilled drivers is higher than that of skilled drivers, which shows that unskilled drivers need longer time to process TEF information. The values of dispersion X and dispersion Y of unskilled drivers are higher than those of skilled drivers, indicating that the horizontal and vertical visual search breadth of unskilled drivers is wider. This is because skilled drivers only pay attention to effective information when driving in a simple landscape environment. The values of the saccade average speed of unskilled drivers are lower than those of skilled drivers, indicating that the skilled drivers have a high information capture rate.

Based on the above overall trend analysis results, the main effect of information quantity of TEF on all drivers and grouped drivers, the interaction between the information amount and driving experience, and the influence of driving experience on driver information processing are analyzed in detail below, respectively.

### Fixation Duration

The fixation duration refers to the period of time a driver takes to process the information, which reflects the complexity of extracting information. The more difficult it is to process the information, the longer the fixation duration lasts. Table 5 shows that when the mean of the fixation duration is described at different levels of information quantity, the fixation duration does not change in a linear way with the increase of the information quantity, and it is different from one level to another.

The Levene’s test was used to explain the error variance of the fixation duration from Z0 to Z5. Results show that the variance is homogeneous, and p equals to 0.120, 0.067, 1.000, 0.053, 0.725, and 0.136, respectively, all of which are greater than 0.05. The repeated measure analysis of variance was used in this study, and results of the Mauchly’s spherical test (*p* < 0.05) show that there is some discrepancy between the actual value and the spherical hypothesis. The Greenhouse-Geisser Epsilon (ε = 0.2) was used to correct the degree of freedom. The main effect of TEF information quantity is found, with *F*_(1.5, 56.2)_ = 26.97 and *p* = 0.000, and the main effect of driving experience is significant, with *F*_(1, 38)_ = 14.03 and *p* = 0.001. Finally, the interaction between the driving experience and the information quantity was detected, with *F*_(1.5, 56.2)_ = 7.43 and *p* = 0.003. It shows that when the information quantity affects the driver’s visual characteristics, driving experience is also an influencing factor. The *post-hoc* test was carried out for six levels of information quantity from Z0 to Z5, and the multiple comparisons results show that there is a significant difference between Z0, the control group and other five levels (*p* < 0.05). Z1 and Z5 have significant differences with other levels (*p* < 0.05), but no significant difference is found among Z2, Z3, and Z4 (*p* > 0.05). The results are shown in Tables 6–8.

**TABLE 6:** Mauchly’s test of sphericity.

Within effect	Mauchly’s W	Approx. chi-square	Df	Sig.	Greenhouse-Geisser	Huynh-Feldt	Lower-Bound
Information quantity	0.000	368.834	14	0.000	0.222	0.237	0.200

**TABLE 7:** The information amount factor main effect and interaction effect of the experience factor.

Within effect test		Type III sum of squares	Df	Mean square	*F*	Sig.
Information quantity	Greenhouse-Geisser	126782.012	1.480	85689.587	26.969	0.000
Information quantity * Driving experience	Greenhouse-Geisser	34959.591	34959.591	1.480	7.437	0.003
Error	Greenhouse-Geisser	178640.761	56.223	3177.367		

**TABLE 8:** The test results of driving experience factor’s main effect.

Between-group effect	Type III sum of squares	Df	Mean square	*F*	Sig.
Driving experience	172265.874	1	172265.874	14.031	0.001
Error	466543.408	38	12277.458		

### Visual Search Breadth

Underwood put forward the concept of visual search breadth to describe the dispersion of the gaze point distribution (Underwood, 2007). The standard deviation of the horizontal (dispersion X) and vertical (dispersion Y) angles of gaze points is defined, respectively, as the horizontal visual search breadth and the vertical visual search breadth. Obviously, the wider the visual search breadth is, the more information a driver can extract. Table 9 shows that there is a difference between every two levels when the mean of visual search breadth is described at different levels of information quantity.

**TABLE 9:** Mauchly’s test of sphericity.

Within effect	Mauchly’s W	Approx. chi-square	Df	Sig.	Greenhouse-Geisser	Huynh-Feldt	Lower-Bound
Information quantity	0.416	31.625	14	0.005	0.739	0.850	0.200

The same method was applied to analyze dispersion X from Z0 to Z5 and results show that the variance is homogeneous and there is a discrepancy between the actual value and the spherical hypothesis. The Greenhouse-Geisser Epsilon (ε = 0.7) was used to correct the degree of freedom. The main effect of TEF information quantity is found, with *F*_(3.7, 140.3)_ = 175.64 and *p* = 0.000, and the main effect of driving experience is significant, with *F*_(1, 38)_ = 13.73 and *p* = 0.001. Finally, the interaction between the driving experience and the information quantity is detected, with *F*_(3.7, 140.3)_ = 25.34 and *p* = 0.000. It shows that when the information quantity affects the driver’s visual characteristics, driving experience is also an influencing factor. The multiple comparison results show that there is no significant difference between the control group and Z1 (*p* = 0.28). However, Z0 and Z1 have significant differences from other four levels (*p* < 0.05), and the values of Z0 and Z1 are lower than the other four levels. The result is shown in Tables 9–11.

**TABLE 10:** The information amount factor main effect and interaction effect of the experience factor.

Within effect test		Type III sum of squares	Df	Mean square	*F*	Sig.
Information quantity	Greenhouse-Geisser	924.492	3.693	250.310	175.638	0.000
Information quantity * Driving experience	Greenhouse-Geisser	133.372	3.693	36.111	25.339	0.000
Error	Greenhouse-Geisser	200.017	140.349	1.425		

**TABLE 11:** The test results of driving experience factor’s main effect.

between-group effect	Type III sum of squares	Df	Mean square	*F*	Sig.
Driving experience	172265.874	1	172265.874	13.735	0.001
Error	12.563	38	0.698		

The same method was applied to analyze dispersion Y from Z0 to Z5, and results show that the variance is not homogeneous. The paired *t*-test was used whose results show that no significant difference is found among Z0, Z1, Z2, and Z5, but there is significant difference between Z4 and other five levels and the value of Z4 is the highest. This indicates that the vertical visual search breadth is narrower at low and high levels of information quantity and the vertical breadth is the largest when the information quantity ranges from 30 to 40 bits/km. Driving experience has a significant impact on the vertical breadth.

### Saccade Average Speed

Saccade average speed refers to the efficiency of capturing and processing information. Table 5 shows that the saccade average speed does not change in a linear way with the increase of information quantity, when the mean of glance speed was described at different levels of information quantity, and it is different from one level to another.

The same method was applied to analyze the saccade average speed from Z0 to Z5, and results show that the variance is homogeneous and there is a discrepancy between the actual value and the spherical hypothesis. The multivariate analysis of variance was used. The main effect of TEF information quantity is found, with *F*_(4, 15)_ = 214.65 and *p* = 0.000, and the main effect of driving experience is significant, with *F*_(1, 38)_ = 162.09 and *p* = 0.000. Finally, the interaction between the driving experience and the information quantity is detected, with *F*_(4, 15)_ = 39.98 and *p* = 0.000. It shows that when the information quantity affects the driver’s efficiency of capturing and processing information, driving experience is also an influencing factor. The *post-hoc* test was carried out for six information quantity levels from Z0 to Z5, and the multiple comparisons results show that there is no significant difference among the control group, Z1 and Z2 (*p* = 0.28), but there is a significant difference among other levels (*p* < 0.05). The result is shown in Tables 12–14.

**TABLE 12:** Mauchly’s test of sphericity.

Within effect	Mauchly’s W	Approx. chi-square	Df	Sig.	Greenhouse-Geisser	Huynh-Feldt	Lower-Bound
Information quantity	0.202	26.274	19	0.002	0.575	0.701	0.250

**TABLE 13:** The information amount factor main effect and interaction effect of the experience factor.

Within effect test		Value	*F*	Hypothesis df	Error df	Sig.
Information quantity	Wilks’ lambda	0.017	214.650	4.000	15.000	0.000
Information quantity * Driving experience	Wilks’ lambda	0.086	39.982	4.000	15.000	0.000

**TABLE 14:** The test results of driving experience factor’s main effect.

between-group effect	Type III sum of squares	Df	Mean square	*F*	Sig.
Driving experience	93132.391	1	93132.391	162.086	0.000
Error	10342.583	38	574.588		

### Result Analysis

The information quantity had a significant impact on a driver’s fixation duration, dispersion X, dispersion Y, and saccade average speed. With the increase of TEF information quantity, drivers need more time to process information. The fixation duration is the lowest in Z0 (0 bits/km), because the gaze duration of a single point is declined when a driver searched surrounding information on monotonous grassland highways. Dispersion X is the lowest in Z0 (0 bits/km), because less attention is paid to the road surface and the visual search breadth is narrower. Dispersion Y is the lowest at the low and high levels of information quantity.

The information quantity from 0 to 10 bits/km is dominant on straight roads of grassland highways. The fixation duration at this level has a significant difference compared to that at other levels, and its mean value is the highest. However, longer fixation duration is not due to the difficulty of processing information. There is no significant effect of Z1 and Z0 on dispersion X and dispersion Y. It is because, with low information quantity and in a monotonous road environment, the driver gazes at the road surface with longer duration. The fixation duration decreases gradually from Z2 to Z4, and the value is the smallest in Z4 (30–40 bits/km). This indicates that with the increase of information quantity, it is more difficult to process the information, and the processing efficiency is the highest in Z4. The search breadth reaches maximum in Z4 (30–40 bits/km), which indicates that Z4 can be a reasonable range used to set TEF.

The driver’s fixation duration reaches the maximum and the saccade average speed reduces rapidly in Z5 (40 bits/km), indicating that it is difficult to process the information, and in this same range a driver catches and processes the information the slowest.

Driving experience had a significant impact on a driver’s fixation duration, dispersion X, dispersion Y, and saccade average speed. There is a difference among Z0, Z1, and Z2, which indicates that skilled and unskilled drivers present different ways of processing information when they are exposed to relatively low information quantity, and skilled drivers have a high information capture rate. The variation of visual search breadth at low and high levels of information quantity is consistent, but visual search strategies of Z2, Z3, and Z4 are different from one another. The information processing efficiency of skilled drivers is the highest in Z4 (30–40 bits/km), nevertheless, the information processing efficiency of unskilled drivers is the highest in Z3 (20–30 bits/km).

## Discussion and Conclusion

Straight roads have typical alignment on grassland highways, which account for 80–90% of the total length of grassland highways. The effect of TEF information quantity on the driving behavior is studied through driving simulation. This study reveals how TEF information quantity on straight roads of grassland highways affects a driver’s eye movements and how the effect changes.

### Appropriate Information Quantity

Most TEFs on straight roads of grassland highways have an information quantity of 0–10 bits/km, which means that 90% of roads have low information quantity (Xie et al., 2014). The variation of the driver’s visual characteristics is noteworthy when the TEFs on straight roads of grassland highways have low information quantity. After considering the variation of the fixation duration and visual search breadth, it can be found that when the information quantity is from 0 to 10 bits/km, the driver’s fixation duration is long and the visual search breadth is narrow. If the driver continuous to drive, his attention will decrease. On grassland highways with low information quantity, the driver receives less necessary stimulation, which may lead to the decrease of his attention, physiological fatigue, and mental fatigue, thus threatening the traffic safety. Since over 90% of TEF on grassland highways have information quantity, it causes trouble to drivers. Hence, traffic engineering facilities on grassland highways should be improved.

The variation of the driver’s fixation duration, visual search breadth, and glance speed when the driver is exposed to the information quantity of over 40 bits/km indicates that excessive TEF information will also bring negative effects. Results show that it is extremely difficult for a driver to process the information, and at this level the visual search breadth is narrow and the information is processed slowly. Although grassland highways have a few roads with high information quantity (over 40 bits/km), this result is still significant and it provides a limit for the maximum TEF information quantity. It is not recommended to have more information than 40 bits/km, otherwise drivers will have more workload and weaker ability to process the information.

It is found that the variation of the driver’s fixation duration, visual search breadth, and saccade average speed, when a driver is exposed to the information quantity from 30 to 40 bits/km, is positive. Results show that at this level the information is processed quickly and efficiently, and the visual search breadth is wide. An information quantity from 30 to 40 bits/km can be used as an appropriate reference to improve traffic engineering facilities on grassland highways.

### Driving Experience

Driving experience has an impact on the driver’s information processing ability and visual search breadth. The duration of process information is longer and the visual search breadth is wider for unskilled drivers. When the information quantity is relatively high and low, the fixation duration is both longer, and dispersion X and dispersion Y are lower. This indicates that the attention of unskilled drivers is more prone to decline on grassland highways while skilled drivers are able to pay more attention to effective information on the road.

It is found that skilled and unskilled drivers have different visual search strategies, information identification, and processing ways when driving on grassland highways. To improve traffic engineering facilities on straight roads of grassland highways, a driver’s experience should be considered.

In this study, according to the current situation of the TEF information environment on the grassland highway, based on the related quantitative theory of information, the physical experiment research method was used, and driver’s eye movement characteristics were explored for the TEF. The design suggestions of TEF on the grassland highway are put forward based on drivers’ visual characteristics. This study can provide a theoretical basis and guidance for the optimization design of TEF on the grassland highway, and enhance traffic safety.

## Data Availability Statement

The original contributions presented in the study are included in the article/supplementary material, further inquiries can be directed to the corresponding author.

## Ethics Statement

Ethical review and approval was not required for the study on human participants in accordance with the local legislation and institutional requirements. The patients/participants provided their written informed consent to participate in this study. Written informed consent was obtained from the individual(s) for the publication of any potentially identifiable images or data included in this article.

## Author Contributions

HL wrote the section of the manuscript. FY, YL, and SZ contributed to data curation and analysis. HL and SX contributed to the manuscript revision. All authors approved the submitted version.

## Conflict of Interest

The authors declare that the research was conducted in the absence of any commercial or financial relationships that could be construed as a potential conflict of interest.

## Publisher’s Note

All claims expressed in this article are solely those of the authors and do not necessarily represent those of their affiliated organizations, or those of the publisher, the editors and the reviewers. Any product that may be evaluated in this article, or claim that may be made by its manufacturer, is not guaranteed or endorsed by the publisher.
